# A comparison of two forms of the continuity equation in the Trifecta bovine pericardial aortic valve

**DOI:** 10.1530/ERP-16-0007

**Published:** 2016-03

**Authors:** John B Chambers, Denise Parkin, James Roxburgh, Vinayak Bapat, Christopher Young

**Affiliations:** Cardiothoracic Centre, Guy’s and St Thomas’ Hospitals, London, UK

**Keywords:** continuity equation, aortic valve replacement, 2D echocardiography, Trifecta

## Abstract

**Aim:**

To compare the classical and simplified form of the continuity equation in small Trifecta valves.

**Methods:**

This is a retrospective analysis of post-operative echocardiograms performed for clinical reasons after implantation of Trifecta bioprosthetic valves.

**Results:**

There were 60 patients aged 74 (range 38–89) years. For the valves of size 19, 21 and 23mm, the mean gradient was 11.3, 10.7 and 9.7mmHg, respectively. The effective orifice areas by the classical form of the continuity equation were 1.4, 1.7 and 1.9cm^2^, respectively. There was a good correlation between the two forms of the continuity equation, but they were significantly different using a *t*-test (*P*<0.00001). Results using the classical form were a mean 0.11 (s.d. 0.18)cm^2^ larger than those using the simple formula.

**Conclusion:**

Haemodynamic function of the Trifecta valve in the small aortic root is good. There are significant differences between the classical and simplified forms of the continuity equation.

## Introduction

The continuity equation is based on the law of conservation of mass and assumes that the stroke volume through a stenotic orifice is the same as upstream from that orifice. For the aortic valve, the effective orifice area is classically estimated from the cross-sectional area in the left ventricular (LV) outflow tract multiplied by the ratio of the systolic velocity integrals on the subaortic pulsed Doppler signal and the transaortic continuous wave Doppler signal. However, a simplified modification uses the ratio of subaortic to transaortic peak velocity. Both forms of the equation were described and compared in the original study (1) introducing the formula to clinical use and both are described in the most recent guidance (2) on the echocardiography of prosthetic valves. Furthermore, effective area using both forms of the equation appears automatically on commercially available echocardiography systems and both are used in clinical practice. It is possible that the form of the equation used affects the resultant effective orifice area estimate independent of other sources of variability, including the level of measurement of the LV outflow tract diameter.

**Figure 1:**
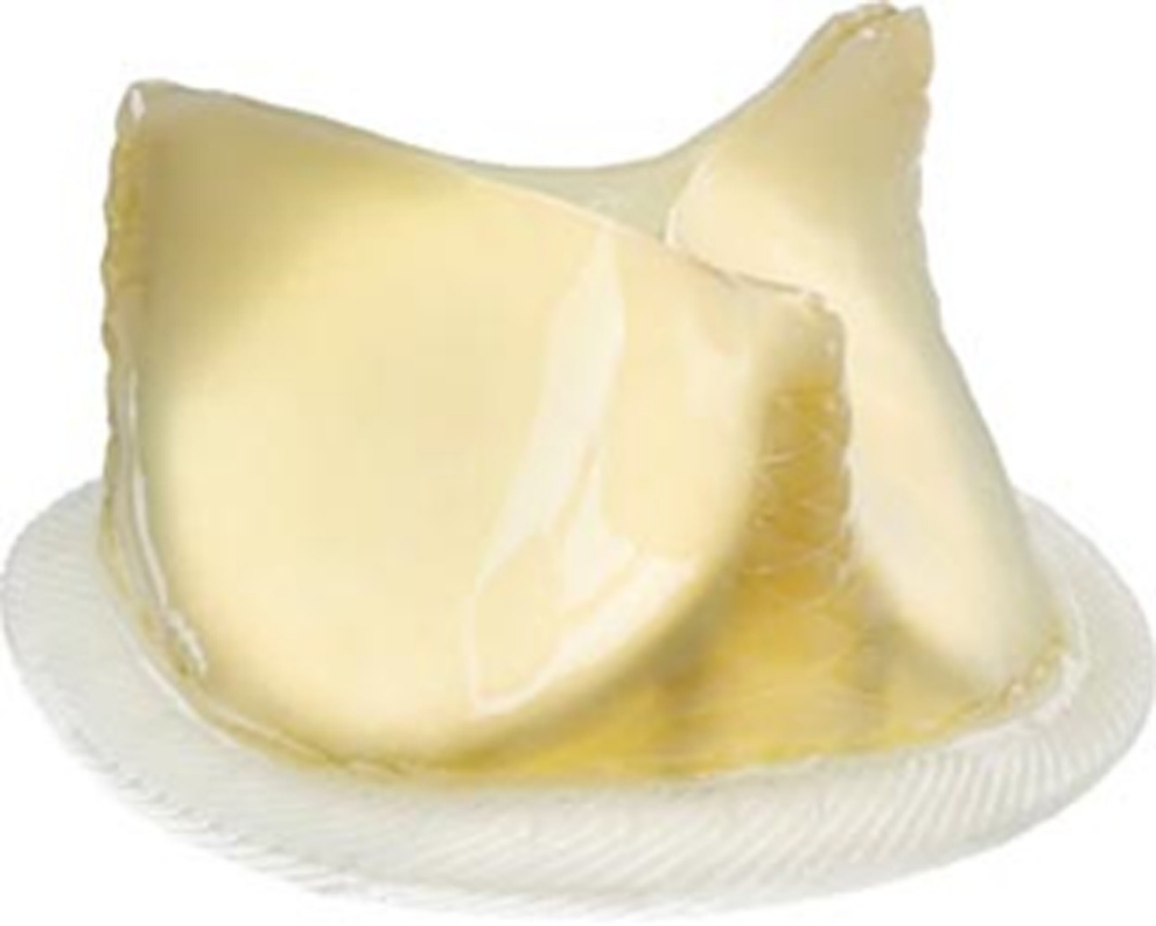
Trifecta valve. The valve consists of a polyester-covered titanium stent to the outside of which is sewn a single pericardial sheet folded to form three cusps.

We compared both forms of the continuity equation as part of the evaluation of the Trifecta valve: a new replacement valve recently introduced to our cardiac surgical practice predominantly for use in patients with small aortic roots. The Trifecta valve is a third-generation replacement valve (Fig. 1) designed to reduce obstruction to flow by the bovine pericardial tissue being sewn to the outside of the polyester-covered titanium and by implanting the valve in a supra-annular position. Gradients at rest are low in the small label sizes between 19 and 23mm (3, 4, 5). However, the reported effective orifice areas (EOA) vary widely, between 1.2 (6) and 1.6cm^2^ (4, 5) for the 19mm size. One reason for this might be the use of different forms of the continuity equation to calculate EOA (1).

We analysed our data for newly implanted, normally functioning aortic Trifecta valves in the 19, 21 and 23mm sizes using the two forms of the continuity equation.

## Method

### Patients

This was a retrospective analysis of post-operative echo­cardiograms performed within 1 year after surgery in patients implanted with a size 19, 21 or 23mm Trifecta valve at one centre (Guy’s and St Thomas’ Hospitals) between 1 March 2013 and 31 January 2015. The study was registered with the hospital audit process as a clinical evaluation (no. 5570).

### Echocardiography

Standard studies were performed as a clinical routine according to standard guidelines (7) by multiple operators on many machines. The continuity equation was calculated retrospectively from measurements obtained on the same waveforms using two formulae (1, 2):

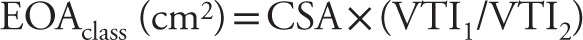


where CSA is the cross-sectional area of the LV outflow tract (incm^2^) calculated assuming a circular cross section using the diameter (in cm) measured close to the base of the aortic cusps. VTI_1_ is the subaortic systolic velocity integral (in cm) and VTI_2_ is the transaortic systolic velocity integral (in cm):

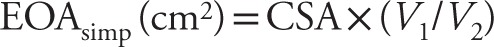


where *V*_1_ is the peak subaortic velocity (in m/s) and *V*_2_ is the peak transaortic velocity (in m/s).

EOA was indexed to BSA. Moderate patient prosthetic mismatch was defined by an indexed effective orifice area >0.65 and <0.85cm^2^/m^2^ and severe mismatch was defined by an indexed effective orifice area <0.65cm^2^/m^2^ (7).

### Analysis

Means and standard deviation values were calculated by valve size. The two forms of the continuity equation were compared using a regression analysis and a paired *t*-test. The differences between the forms were calculated and compared using a Bland–Altman analysis. The numbers of patients with moderate patient–prosthesis mismatch were compared using a *χ*^2^ test.

## Results

**Figure 2:**
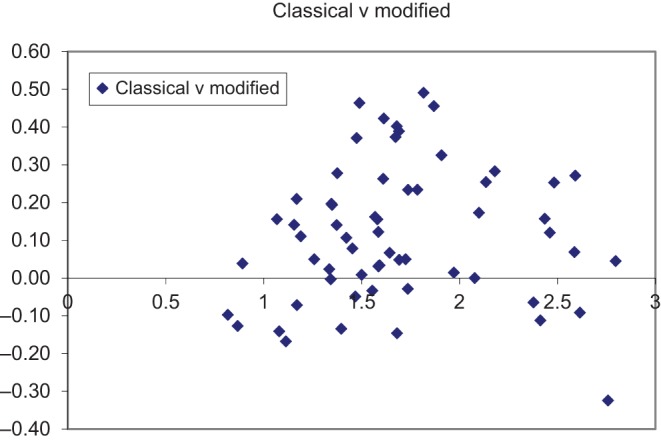
Bland–Altman plot comparing agreement between the two forms of the continuity equation.

There were 61 patients, but one was excluded as a result of finding moderate paraprosthetic regurgitation. The study population was therefore 60 patients aged 74 (range 38–89) years at the time of surgery. Echocardiography was performed at a mean 63 days (range 2–342) after surgery. Concomitant procedures were performed in 20patients (Table 1). Haemodynamic function is given in Table 2. There was a good correlation between the results using the two forms of the continuity equation (*r*^2^ 0.9), but they were statistically highly significantly different using a *t*-test (*P*<0.00001). The orifice area value using the classical formula was a mean 0.11 (0.18)cm^2^ larger than that using the simplified formula (Fig. 2).

**Table 1:** Preoperative demographic data.

	**Trifecta valve size**			**Whole group** (*n*=60)
	19mm (*n*=10)	21mm (*n*=23)	23mm (*n*=27)	
Male:Female	0:10	6:17	20:7	26:34
Age (s.d.)	77 (6)	76 (9)	72 (9)	74 (9)
Concomitant procedures				
CABG	3	5	9	17
MVR	1	0	1	2
Aortoplasty	0	0	1	1
LV function				
EF > 40%	10	23	26	59
EF<30–40%	0	0	1	1
EF<30%	0	0	0	0

**Table 2:** Postoperative haemodynamic results.

**Result**	**Trifecta valve size**		
	19mm (*n*=10)	21mm (*n*=23)	23mm (*n*=27)
Forward flow			
*V*_max_	2.2 (0.3)	2.3 (0.4)	2.1 (0.4)
Mean gradient	11.3 (2.9)	10.7 (4.1)	9.7 (4.8)
EOA classical	1.4 (0.6)	1.7 (0.5)	1.9 (0.4)
EOA simplified	1.3 (0.5)	1.6 (0.5)	1.8 (0.4)
EOAi classical	0.8 (0.4)	1.0 (0.3)	1.0 (0.2)
EOAi simplified	0.8 (0.3)	0.9 (0.3)	0.9 (0.2)
Regurgitation through the valve			
None	8 (80%)	14 (61%)	21 (78%)
Trivial	2 (20%)	6 (26%)	4 (15%)
Mild	0	3 (13%)	2 (7%)
Paraprosthetic regurgitation			
None	10	20 (87%)	25 (93%)
Trivial or mild	0	3 (13%)	2 (7%)

There was trivial or mild regurgitation through the valve in 17 (28%) patients (Table 2) and in a paraprosthetic position in 5 (8%) patients. Moderate patient–prosthesis mismatch was present in ten (17%) patients using the classical form of the continuity equation and in 20 (33%) patients using the simplified form. This was statistically significant (*χ*^2^ statistic 4.6, *P*=0.03). Severe patient–prosthesis mismatch was present in 5 (8%) patients each respectively.

## Discussion

This study showed good haemodynamic function of the Trifecta valve in the small aortic root. The simplified form of the continuity equation gave significantly lower values for EOA than the more correct classical form.

The haemodynamic data are broadly similar to the literature (3, 4, 5, 8, 9) in which the reported mean gradients are 9.3–14mmHg for the size 19mm valve, 7.8–12mmHg for the size 21mm valve and 6.9–11 mmHg for the size 23mm valve. There is little comparative work, but others have suggested similar (9, 10) and in some cases better (10, 11) function than other biological valves particularly for size 19mm valves (12, 13, 14). The proportion with moderate patient–prosthesis mismatch using the classical form of the continuity equation, 17%, compares favourably with the reported prevalence of 20–70% in all types of replacement valves (7). The proportion of severe patient–prosthesis mismatch, 8%, was similar to the reported prevalence of 2–11% (7).

The differences in EOA depending on the form of the continuity equation are potentially important in the comparison of different studies. The form of the equation was not stated in previously published work, and the EOA for size 19mm valves was 1.2cm^2^ (6), 1.4cm^2^ (8, 9) and 1.6cm^2^ (4, 5), consistent with the difference of 0.11cm^2^ obtained between the two forms of the equation within this study. The continuity equation is based on the law of conservation of mass and relies on the stroke volume not changing between the subaortic and transaortic positions. Stroke volume is calculated using the systolic velocity integral, and the ratio of velocities is only a reasonable approximation if the subaortic and transaortic waveforms are of the same shape, which may not be true. At EOAs below approximately 1.0cm^2^, the simplified form tends to give larger values (1) whereas above 1.0cm^2^, the classical form tends to give larger values (15).

## Limitations

The number of patients at each valve size was small, although the differences between the two forms of the continuity equation were consistent and likely to be genuine.

## Conclusion

Haemodynamic function of the Trifecta valve in the small aortic root is good. There were significant differences between the effective orifice areas obtained using the classical and simplified forms of the continuity equation. The classical form should be preferred but, as a minimum, it is important to state which form is used and to maintain the same form in serial studies.

## Declaration of interest

The authors declare that there is no conflict of interest that could be perceived as prejudicing the impartiality of the research reported.

## Funding

St Jude Medical funded the research nurse for the period of this study but played no part in the design, execution or writing of the project.
